# Urethral Duplication With Dorsal Chordee: A Case Report and Literature Review

**DOI:** 10.7759/cureus.55444

**Published:** 2024-03-03

**Authors:** Alhareth A Baarimah, Latif Dar, Saeed Alshahrani, Khaled Aldhabaan

**Affiliations:** 1 Department of Pediatric Urology, Khamis Mushayt Maternity and Children Hospital (KMMCH), Khamis Mushayt, SAU; 2 Department of Pediatric Urology, Abha Maternity and Children Hospital (AMCH), Abha, SAU

**Keywords:** penile deformity, case report, pediatric urology, chordee, urethral duplication

## Abstract

Urethral duplication is a diverse spectrum of disease having multiple anatomic variants. The clinical presentation varies from being asymptomatic to recurrent urinary tract infections. A high level of clinical suspicion and awareness among primary caregivers is needed to make a proper diagnosis. All patients presenting with any sort of penile deformity or abnormality of the urinary stream should be evaluated to rule out this condition. In this case report the patient had presented with the urinary stream being directed towards his abdomen due to abnormal dorsal curvature of the penis which was due to tethering of the accessory urethra.

## Introduction

Although the first case of urethral duplication was reported very early by Aristotle, it is such a rare condition that till now only less than 300 cases have been reported [1,2]. It is a diverse spectrum of disease defined as the co-existence of two musculo-epithelial urethral channels covered by the urinary type of epithelium [2,3]. The condition has a high male preponderance with only a few case reports in females. It is broadly classified as complete and incomplete duplication with a number of anatomic variations [4]. Associations such as renal and bladder duplications have been reported, dorsal chordee is rare [5-7]. Clinical presentation varies, and management depends upon the anatomic variant. Radiological imaging is important in defining the anatomy and deciding the therapeutic approach. A four-year-old patient with incomplete urethral duplication associated with dorsal chordee is presented, with a literature review describing the management.

## Case presentation

**Figure 1:**
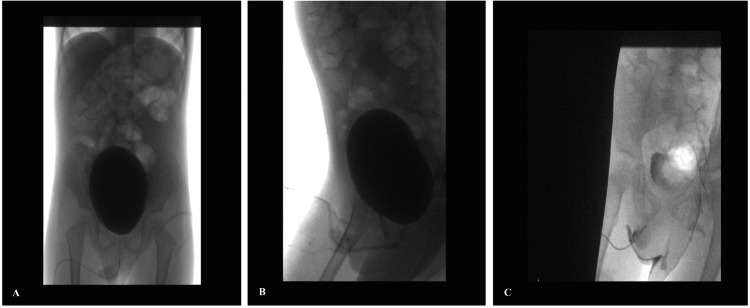
Cystourethrogram A, B: Cystourethrogram was done by catheterizing the ventral urethral opening and the urinary bladder showed normal appearance and contour. C: The accessory urethra was injected by contrast which demonstrated a blunt ending with no evidence of communication with the urinary bladder or to the ventral urethra.

A four-year-old boy was referred to pediatric urology with complaints of a curved penis and the urinary stream directed towards his abdomen. No other urinary symptoms were noticed. On evaluation, the patient was found to have an average-sized circumcised penis with a mild degree of Dorsal chordee. Two urethral openings were found. One was normally located over the glans, and the other one was found over the dorsum about 1 cm proximal to the normal meatus. The boy was asked to micturate. He passed urine from the normal meatus, but the stream was directed up towards his abdomen. The scrotum was looking normal with both testes palpable. The ultrasound abdomen showed normal kidneys and bladder. Micturating cystourethrogram was done by catheterizing the ventral urethral opening under sterile conditions. About 200 cc of diluted water-soluble contrast was dripped by gravity into the bladder which revealed a urinary bladder of normal appearance and contour, no wall thickening or trabeculations were seen. The voiding phase showed a normal-appearing urethra with no communications (Figure 1a, 1b). This was followed by cannulation of the dorsal urethral opening using a 4F feeding tube. Pure contrast was injected which demonstrated a blunt ending tubular structure with no evidence of communication with the urinary bladder or to the ventral urethra (Figure 1c). A diagnosis of incomplete duplication of the urethra was made and the patient was prepared for surgery.

**Figure 2:**
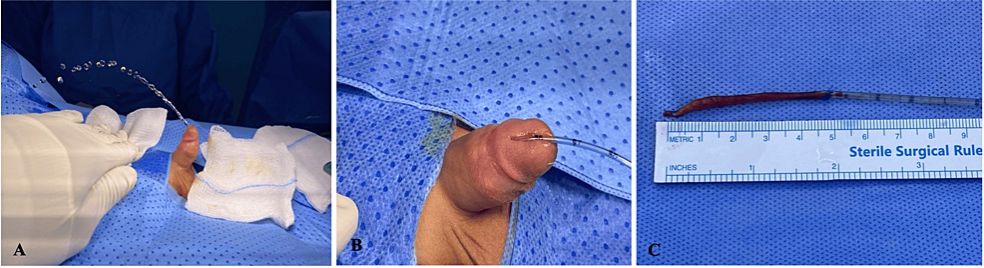
The accessory urethra A: Examination under anesthesia confirmed the presence of chordee and urinary stream directed towards the abdomen. B: The accessory urethra was cannulated using a 6F feeding tube. C: The dorsal urethra was totally excised, and it was 5 cm in length.

Examination under anesthesia confirmed the presence of double urethra and chordee (Figure 2a). Cystourethroscopy was done, which did not show any evidence of communication of the dorsal urethra to the urinary bladder or to the ventral urethra. The normal urethra was catheterized using an 8F Foley catheter. The accessory urethra was cannulated using a 6F feeding tube (Figure 2b). Degloving of the penis was performed in the usual manner. The accessory urethra was dissected and excised in toto up to its blind ending (Figure 2c). A significant tethering of the accessory urethra with the corporal tissues was observed which was leading to the dorsal chordee. The excision of the accessory urethra corrected this deformity which was confirmed by the erection test. Care was taken about the preservation of the neurovascular bundle.

**Figure 3:**
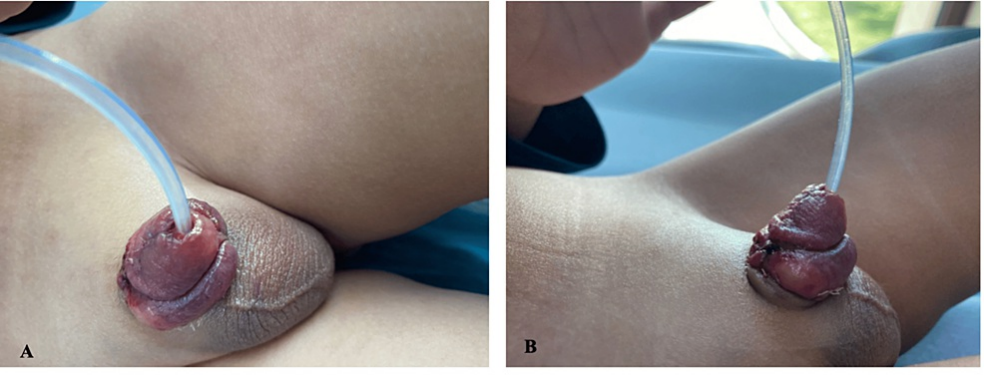
The follow-up A: The boy in the clinic on the third post-op day with a single urethra. B: The dorsal chordee has been resolved and no more penile deformity.

The post-operative period was uneventful, and the patient was discharged home the same day. The catheter was removed on the third post-op day in the clinic (Figure 3). On follow-up, the patient was passing urine in a good, single, straight stream without any penile deformity.

## Discussion

Urethral duplication is such a rare condition that only less than 300 cases have been reported since it was first mentioned by Aristotle and Vesalius [1,2]. To date, whatever is known about this condition comes from case reports and literature reviews. It is mainly seen in boys; girls are rarely affected. Most of the cases reported so far have been asymptomatic [3,4]. The presentation with dorsal chordee is rarely reported. Our patient had no complaint other than the penis being bent upwards and the urinary stream was directed towards the abdomen of the patient. This presentation is rarely mentioned in the literature before. In Nnabugwu et al.'s review of 231 cases of urethral duplication, 15 patients had dorsal chordee [5].

Urethral duplication is not a uniform entity, it has diverse anatomic variations. Epispadias, hypospadias, fusiform and Y-type are the main anatomic variants. Various classification systems have been proposed. It has been divided into two basic types: complete and incomplete. The complete type begins in the urinary bladder and ends in an external opening while the incomplete one does not originate from the bladder; the internal end may be blind or may communicate with the urethra. Effmann proposed a classification system which received wide acceptance among clinicians and researchers. He classified urethral duplication into three types: Type I with subtypes IA and IB is the incomplete urethral duplication. Type II represents the complete duplication with its subtypes. Type III is the urethral duplication associated with caudal duplication (i.e., duplication of the bladder). Our case was Type IA in which the accessory urethra is a blind-ending channel opening on the dorsal surface of the penis in midline, without communicating with either the bladder or urethra [4-7].

Radiological imaging is of utmost importance, not only to make a diagnosis but to define the anatomic type and course of the accessory urethra. We used ultrasound to rule out associated renal anomalies. Micturating urethrogram and retrograde urethrogram were used for a good visualization of the normal and accessory urethra.

The surgical management should be individualized as no single surgical technique can be offered to a spectrum of diverse anatomic variants. The complete type of defects may need multiple surgical interventions while an asymptomatic incomplete type may be left untreated [3-7]. In our case, as the family of the patient was worried about the shape of the penis, we offered them surgical correction. The accessory urethra was completely excised and the penile curvature was corrected. On follow-up, the patient was passing urine in a single straight stream with a good cosmetic outcome.

## Conclusions

Urethral duplication is not a uniform entity, it has diverse anatomic variations. Keeping in view the diversity and anatomic variations in urethral duplication, a high level of suspicion is needed for diagnosis. All patients with any sort of penile deformity and abnormality of the urinary stream should be evaluated to rule out urethral duplication. There is no single surgical technique that can be offered to all the anatomic variants of urethral duplication. The management needs to be individualized. In some cases, only a simple excision of the accessory urethra leading to chordee can correct the abnormality, while multiple surgical interventions are required in some of the complex complete defects.
